# Employment Legal Framework for Persons with Disabilities in China: Effectiveness and Reasons

**DOI:** 10.3390/ijerph17144976

**Published:** 2020-07-10

**Authors:** Yuling Hao, Peng Li

**Affiliations:** 1 School of Public Affairs, Xiamen University, No. 422, Siming South Road, Xiamen 361005, China; 2 Graduate Institute for Taiwan Studies, Xiamen University, No. 422, Siming South Road, Xiamen 361005, China; 3 Collaborative Innovation Center for Peaceful Development of Cross-Strait Relations, Xiamen University, No. 422, Siming South Road, Xiamen 361005, China; 4 Taiwan Studies Center, Xiamen University, No. 422, Siming South Road, Xiamen 361005, China

**Keywords:** persons with disabilities, disability model, employment, antidiscrimination, employment quota scheme, legal framework

## Abstract

In order to promote the employment of persons with disabilities, two dominant legal approaches—anti-discrimination legislation based on the social model of disability and an employment quota scheme based on the medical model—are usually employed on a nation-state basis in disability policies. This article systematically examines the reasons why both the anti-discrimination and employment quota scheme legal frameworks have limited effectiveness in promoting employment of persons with disabilities in China. We found that the lack of a definition of disability, the lack of a definition of discrimination, and the absence of effective enforcement mechanisms are the reasons for poor outcomes of the anti-discrimination legal framework. For the employment quota scheme, conflicts between the mainstream labor market legal framework and the quota scheme legal framework have prompted employers to pay penalties rather than hire persons with disabilities. China should address these issues in the current legal system in the short term. Meanwhile, the CRPD should be more strongly emphasized in China. This article argues for the human rights model espoused by the CRPD, instead of the medical model, to develop a coherent and sustainable disability legal framework for promoting participation of persons with disabilities, rather than focusing on viewing them as recipients of care.

## 1. Introduction

In China, according to the most recent statistics, based on the 2006 Second National Sample Survey on Disability, there were 85 million people, or about 6.4% of the total population living with some form of disability. However, China employs a narrow medical definition of disability. Based on the World Report on Disability, which adopted the disability definition of the International Classification of Impairment, Disability and Health (ICF), the average prevalence rate of disabilities was 15% worldwide in 2011 [1]. Were China to use the international measure in the estimates of the disabled population, it would be undoubtedly be significantly higher than the officially reported number.

To be sure, the situation of persons with disabilities in China has improved significantly in recent years, especially in the context of the Targeted Poverty Alleviation Strategy adopted by the national government in 2014, in which people with disabilities and their family members, as a core group of the poor, are included in and supported by the majority of public poverty alleviation resources. According to data collected via the National Household Income Survey of Persons with Disability in 2017, half of the disposable household income of persons with disabilities were from public transfer, while that was just 18.3% of the national disposable household and per capita income in the same year [2]. Meanwhile, the status of participation of persons with disabilities in the workplace remains very poor. Lower rates of employment have been persistently observed for persons with disabilities. A 2009 study conducted by the International Labor Organization (ILO) showed that China’s economic cost of employment exclusion of people with disabilities accounted for 4.3% of the GDP, almost 111.7 billion dollars in 2006 [3]. Although there are an estimated 85 million people with disabilities, there were only 34 million people voluntarily registered as having a disability in 2019 and only about 4.31 million of them were engaged in work. About 50.4% of those who were working were still engaged in agriculture, growing and breeding [4].

Since 2000, some Chinese scholars have examined the reasons for high unemployment and vulnerable employment of persons with disabilities. A study based on data from the 2006 Second National Sample Survey on Disability found that health condition, education level, social security and local economic development impacted access to employment for persons with disabilities [5]. An analysis of the association between economic growth and employment of persons with disabilities showed that rapid economic growth played little role in promoting the employment of persons with disabilities [6]. A research study from the employment stakeholders’ perspective argued that employers were focused on economic rationalities, while national responsibility was relatively absent in the employment of persons with disability [7].

However, to date, both the international and Chinese literature have shown little interest in the employment legal framework for persons with disabilities in China. A legal framework can create social barriers by creating classifications based on competency or abilities; therefore, it can also foster equality and inclusion [8]. A well-established legal framework is the foundation of an effective and well-functioning disability policy system. From the legal perspective, understanding the place of disability in society has been a very important part of Disability Studies [9]. This article seeks to systematically discuss why both of the dominant legal approaches—the anti-discrimination and employment quota scheme legal frameworks, which are based on different concepts of disability, have limited effectiveness in promoting employment of persons with disabilities in China. The remainder of the article is organized as follows: Section 2 outlines the theoretical underpinning of the employment legal framework for persons with disabilities. Section 3 examines the poor outcomes of the anti-discrimination legal framework. Section 4 presents the weak impact of the employment quota scheme legal framework. Section 5 offers our conclusions.

## 2. The Theoretical Underpinning of the Disability Employment Legislation

The social construct nature of disability is a foundational concept in disability-related legislation [10]. This section examines the theoretical understandings about disability and employment legislation that have been formulated based on these theories.

### 2.1. Explaining Disability in the International Context

Disability is a “complex, dynamic, multidimensional and contested” concept. Historically, disability has been framed as a medical issue. The medical model of disability emerged from doctors and scientists who replaced religious leaders as the ones who have the power or authority to establish definitions in society [11]. The model regarded disability as an impairment, indicating that it was the individual’s defect and problem [12]. In order to promote the inclusion of people with impairments, disability needed to be medically cured or rehabilitated [13].

In the 1970s, the medical model was strongly criticized for ignoring the psychology and social elements of disablement [14]. Based on these criticisms, the World Health Organization (WHO) published the International Classification of Impairments, Disabilities and Handicaps (ICIDH) in 1980. ICIDH was intended to break the control of a medical explanation of disability. It classified disablement factors into three parts, i.e., impairment, disability, and handicap, which were all related to the consequence of a disease. Impairment was defined as “concerned with abnormalities of body structure and appearance and with organ or system function, resulting from any cause”; disability reflected “reflecting the consequences of impairment in terms of functional performance and activity by the individual”; and handicap was described as “concerned with the disadvantages experienced by the individual as a result of impairments and disabilities” [15]. The significance of ICIDH was that it extended the medical model of disability into the social realm by introducing the dimension of handicap, which “reflect interaction with and adaption to the individual’s surroundings” [15]. However, the disablement process presented in the ICIDH was characterized as a unidirectional sequence. According to this logic, both disability and handicap arose as a direct consequence of individual impairment, so impairment was the precondition of disability and handicap.

In the 1980s, another form of criticism of the medical model, the social model of disability, rose to prominence. This model argued that the disablement logic of ICIDH was not an alternative, but further reinforced the medical model by tracing the root of disability to impairment [16]. Especially the dimension of handicap was at issue, since it was noted that “a person’s functional limitations (impairments) are the root cause of any disadvantages experienced and these disadvantages can therefore only be rectified by treatment or cure” [17]. Therefore, this framework failed to analyze disability as resulting from social and environmental forces.

The social model of disability rejected the presumption embraced in ICIDH that disability was derived from bodily limitations [18]. Instead, it differentiated between impairment and disability. This model argued that disability was created by society rather than by the body. It was a social construct created through discrimination and oppression [19]. Oliver, one of the founding fathers of the social model of disability, described disability as “culturally produced and socially constructed” [16]. According to the social model, policies’ response to disability should be designed to remove the physical, social and attitudinal barriers. The social model served as a stepping stone in struggles for civil rights reform and anti-discrimination laws in many countries [20]. However, this model also has attracted criticism as strong as the criticism of the medical model of disability. These criticisms include three main aspects: neglecting the experience of impairment; the impairment/disability dualism; and the issue of identity [21].

In 2001, WHO published a revision of ICIDH, which was termed the International Classification of Functioning, Disability and Health (ICIDH-2 or ICF) and was based on a workable compromise between the medical and social approaches of disablement [1]. ICF acknowledges that disability is neither a merely medical issue nor a merely social issue. Rather, it is the outcome of interactions between individual health conditions and contextual factors, and thus this is characterized as the interactive model of disability. Accordingly, ICF is structured in two parts, Functioning and Disability, and Contextual Factors.

Functioning and Disability in ICF describes personal health conditions. It covers two components: body functions and structures, and activities and participation. These components are used in two ways. First, Functioning, as the umbrella term of health and health-related states, focuses on positive aspects. Second, disability as the umbrella term of impairment, activity limitation or participation restriction, is used to indicate problems. Impairment means “problems in body function or structure such as a significant deviation or loss”; activity limitation denotes “difficulties an individual may have in executing activities”; and participation restriction is described as “problems an individual may experience in involvement in life situations” [22]. Therefore, impairment, activity limitation and participation restriction are reflected by the health condition of the body, the whole person and the social level, respectively.

The section on Contextual Factors in the ICF constitutes the main difference from the previous classification of the ICIDH, as it acknowledges the critical role of the environment in creating disability. This part includes two components: environmental factors and personal factors. Environmental factors refer to “the physical, social and attitudinal environment in which people live and conduct their lives” which can either facilitate or hinder the functioning of persons with impairment [22]. Personal factors are those independent of individual health conditions but may have an influence on one’s functioning such as gender, age, education, social background, etc.

In brief, the ICF presents disability as including difficulties encountered in any or all three areas of functioning, including body functions and structures, activities and participation, and contextual factors (environment factors and personal factors) [1]. A policy response to disability should focus on addressing factors in all three areas. The ICF has provided an international common language and tool for understanding, research, surveillance, reporting, describing, measuring and comparing health and disability-related topics in the world.

In 2006, the Convention on the Rights of Persons with Disabilities (CRPD) was adopted. It conceptualizes disability from the ICF perspective. Disability is expressed as “resulting from the interaction between persons with impairments and attitudinal and environmental barriers that hinder their full and effective participation in society on an equal basis with others” [23]. The CRPD recognizes that disability is a human rights issue. This treaty aims to “promote, protect and ensure the full and equal enjoyment of all human rights and fundamental freedoms by all persons with disabilities, and to promote respect for their inherent dignity” [23].

### 2.2. Understanding Disability in the Chinese Context

In China, Confucianism has commonly been regarded as the core of Chinese culture. It stresses that the perfect society is one in which “the elder, the infirm, the sick and the disabled are exempted from part or whole social obligations and are cared for by the society which has adopted this as the country’s national governing philosophy” [24]. In current days, this thinking still has a great impact on policy making and awareness in society about disability. During the five-thousand-year history of China, disability was firstly termed as “Fei Ji”, Fei means useless and Ji means disease. Then, it was changed to “Can Fei”, Can means handicap and Fei means useless. Since the 1980s, the term for disability has been modified to “Can Ji” that refers to handicap and disease [25]. Tracing these conceptual and semantic changes about disability, demonstrates that the medical model of disability remains popular in the understanding of disability in China. The Law of the PRC on the Protection of Persons with Disabilities of 1990 (Order No. 36 [1990] of the President) gave the official definition of a disabled person, in Article two:

A disabled person is referred to as those who suffer from abnormalities of loss of a certain organ or function, psychologically or physiologically, or in anatomical structure and has lost wholly or in part the ability to perform an activity in the normal way.

[26]

In 1995, China began the official registration for persons with disabilities by introducing the Disability Identification Card. Meanwhile, the China Disabled Persons’ Federation issued the China Practical Disability Determination Standard (CDPF, No. 61 [1995] (hereafter “the Standard”) as the identification criteria for persons with disabilities. The Standard is still in effect today without any amendment. According to the Standard, disabilities are classified into seven categories—visual, hearing, speech, physical, intellectual, psychological, multiple disabilities and/or other disabilities. The category and degree of one’s disability is determined by a designated doctor according to the impairments that are listed and regulated in the Standard [27]. Since then, this definition of disability has been used in the majority of laws and policies related to persons with disabilities. The Disability Certification Card is almost the only way that persons with disabilities are identified for allocation of policy resources.

Summarizing the analysis above, the definition of disability, as well as the identification of persons with disabilities in China are based on the concept of disability in the ICIDH. China signed the UN Convention on the Rights of Persons with Disabilities (CRPD) on 30 December 2007 and ratified it on 3 May 2018. However, as most of the States Parties of CRPD, the paradigm shift to the human rights model has yet to be reflected in implementation [19].

### 2.3. Two Disability Employment Legislative Approaches

The purpose of disability employment legislation is to promote the participation of persons with disabilities in work. Individuals who experience a “precarious relationship with the labor market” face high possibilities of barriers related to social and political participation, as well as necessities integral to the quality of life [28]. Thus, disability policies in welfare state countries have experienced a convergent reform path that is broadly shifting in focus from passive income maintenance to employment incentives and reintegration policies, in particular in the OECD countries during the last four decades [29].

In order to promote the employment of persons with disabilities, two dominant legal approaches are usually employed on a nation-state basis in disability policies, i.e., anti-discrimination legislation and an employment quota scheme, and these are based on different models of disability [30]. The employment quota scheme is inspired by the medical model of disability which views disability as a having a medically determined status. The assumption of this legal approach is that disability, as the individual’s defect and problem, results in him/her unable to work in a conventional way. Accordingly, those who are labeled as disabled have no ability to equally compete for jobs on their own merits with their counterparts without disabilities in the open labor market. Thus, it is necessary to create the quota scheme as a legislative intervention to promote employment of persons with disabilities [31]. Anti-discrimination legislation, on the other hand, is inspired by the social model of disability [20]. The assumption of this legal approach is that disability is the product of inequality and discrimination. Thus, this approach demands changes in society to include persons with disabilities in the open labor market by way of anti-discrimination legislation, rather than segregate them in, and thus legitimizing, special facilities. In contrast to the employment quota scheme that emphasizes the inability of people with disabilities, anti-discrimination legislation focuses on their competence and capacity [32].

In China, anti-discrimination legislation and the employment quota scheme have been integral parts of disability employment policies. In 1990, the employment quota scheme was introduced by the Law of the PRC on the Protection of Persons with Disabilities (Order No. 36 [1990] of the President). In terms of anti-discrimination legislation, China has already prohibited disability-based discrimination in several laws. However, as a State Party of CRPD, discrimination against persons with disabilities in the workplace is still prevalent. This is evident in that the employment offered in the employment quota scheme is only of symbolic value due to deep-rooted discrimination [33]. In fact, discrimination is just one aspect; the other important aspect contributing to the exclusion of persons with disabilities from employment is the conflict between the mainstream labor market legal framework and the special employment legal framework for persons with disabilities, which has been ignored by academic research.

## 3. Poor Outcomes of the Anti-Discrimination Legal Framework

In this section, the anti-discrimination legal framework for employment of persons with disabilities in China is first reviewed. Then, the reasons for the poor outcomes of this legal framework are analyzed.

### 3.1. The Anti-Discrimination Legal Framework

The anti-discrimination legal framework for employment of persons with disabilities in China is composed of policies at both the international and national levels. At the international level, China signed the CRPD in 2007, and ratified it in 2008. Article 27 of the CRPD places a prohibition on employment discrimination and provides rights to equal remuneration, reasonable accommodation, favorable and safe working conditions, systems for redressing grievances, union participation, and access to technical and vocational guidance and training [23].

At the national level, there are two important pieces of legislation. One is the Law of the PRC on the Protection of Persons with Disabilities (2008 Amendment) (Order No. 3 [2008] of the President), and the other is the Regulation of the PRC on the Employment of Persons with Disabilities (Order No. 488 [2007] of the State Council) adopted in 2007.

The Law of the PRC on the Protection of Persons with Disabilities (2008 Amendment) (Order No. 3 [2008] of the President) was the first specific law for the protection of persons with disabilities in China. Article 38 Paragraph 2 of this law states that:

*No discrimination against persons with disabilities shall be practiced in the employment, promotion, determination of technical and professional titles, remunerations, welfare, rest and vacation, social insurances,* etc.

[34]

The Regulation of the PRC on the Employment of Persons with Disabilities is the first special regulation on the employment of persons with disabilities in China. Article 13 of this regulation also has the same provision as that of Article 38 Paragraph 2 in the Law of the PRC on the Protection of Persons with Disabilities (2008 Amendment) (Order No. 3 [2008] of the President) [35].

Although there are clear provisions in the legal framework, disability-based discrimination is still common. As the analysis above demonstrates, anti-discrimination legislation is developed based on a social model of disability, which locates the problem of disability in society rather than the individual person. Therefore, the legislative purpose is to address inequality and discrimination that arise from misperceptions and stereotypes of persons with disabilities. Accordingly, two basic questions must be raised: How is disability defined in order for rights/protections to arise? What forms of non-discrimination protection are provided for? In other words, an anti-discrimination legislation needs to define disability and discrimination clearly [36]. In addition to a wider definition of disability and a strong definition of discrimination, the law needs to provide clear and effective enforcement mechanisms through which persons with disabilities, individually or as a group, should play a major role [37]. Below, the reasons for poor outcomes of the anti-discrimination legal framework in China are explored explicitly from these three aspects.

### 3.2. Reasons for Poor Outcomes of the Anti-Discrimination Legal Framework

A first reason is the lack of a disability definition in the current legal framework in China. How to define disability in legislation has been highly debated around the globe [38]. Although the WHO has effectively developed the ICF framework, there is no universal international legal definition of disability. In the international context, two—one narrow and one wider—approaches are employed to define disability in the anti-discrimination legislation. The narrow approach defines disability based on a present impairment which reflects the thinking of the medical model, and persons who fall under such a definition can be protected against discrimination. The wider approach defines disability based on a past, present, future or assumed impairment-related condition which reflects disability as a social construct, and persons who meet such a definition can receive discrimination protection [36]. A 2005 research study found that there were more than 40 countries who have adopted disability discrimination laws. While the narrow approach has still prevailed, the trend has been towards the wider approach among these countries [37].

In the anti-discrimination legal framework, it is necessary to define disability using either a narrow or a wider approach in order to identify who is protected. However, such a clear definition is missing in the Chinese anti-discrimination legal framework. Besides the provisions as noted above, there is no other guidance for interpretation of the provisions. As a result, even if a person experiences disability-based discrimination, he/she cannot be protected due to the lack of a legal provision for identifying whether she/he has a disability. For example, in the recruitment process for civil servants, candidates must pass a mandated health examination according to the General Standard for Civil Service Recruitment Health Examination [39]. Further, these health examinations have also been adopted by State Owned Enterprises and most large and middle-sized private enterprises. The General Standard is stringent and thus most candidates with impairments fail the examination. This is one of the main reasons that the majority of disabled workers are confined to informal and low quality employment. This phenomena has been discussed in some influential social media in China [40].

A second reason is that there is no clear definition of discrimination. According to a study by Degener in 2005, definitions of discrimination in anti-discrimination legislations range “from unjustified differentiation to direct or indirect unfavorable treatment, to detailed lists of discriminatory practices” around the world [41]. Generally speaking, three forms of disability discrimination are categorized, namely direct discrimination, indirect discrimination and the legal duty of employers to provide reasonable accommodation in the workplace [41]. An instance of direct discrimination occurs when one person with an impairment is excluded because of differential treatment such as categorical exclusion, while an indirect discrimination arises when uniform treatment impacts them differently. Reasonable accommodation discrimination exists when an employer fails to provide reasonable accommodation for the known physical or mental limitations of a person with a disability, unless the employer can show the accommodation would impose an undue burden [42]. In some counties, discriminatory practices are detailed listed in the legislation. For instance, in the Americans with Disabilities Act Amendments Act of 2008 (PL.110-325(S3406)), the definition of “discrimination” sets out that a discriminatory action is one that less favorably treats a person “on the basis of [his/her] disability”, and a number of discriminatory practices are detailed listed [43]. In China, the definition of discrimination is not specified in the anti-discrimination legal framework. The CRPD Committee observed this point regarding China:

While commending the legal prohibition of disability-based discrimination in the state party, the Committee is concerned about the lack of a comprehensive definition of discrimination against persons with disabilities.

[33]

In 2016, some scholars expressed their arguments in the China state-owned newspaper, the Legal Daily on this issue [44]. They called for introducing a specific employment discrimination law to solve the pervasive disability-based discrimination in the workplace. However, there has been no substantive progress so far.

A third reason is the absence of effective enforcement mechanisms. There are two provisions regarding enforcement mechanisms in the Regulation of the PRC on the Employment of Persons with Disabilities (Order No. 488 [2007] of the State Council).

Article 59 provides that:

(i) Where any of the legal rights and interests of a person with disabilities is violated, he or she may file a complaint with the disabled persons’ organizations (DPOs). The DPOs shall protect legal rights and interests of him or her and have the right to require a relevant department or entity to investigate and deal with the case. These entities shall legally do it and make a reply.

(ii) Where a person with disabilities needs help in the protection of his legal rights and interests through litigation, the DPOs shall give support to him or her.

(iii) The DPOs shall have the right to require the relevant departments to legally investigate and deal with any violations of the interests of specific group of persons with disabilities.

[35]

Article 60 states that:

If the legal rights and interests of a person with disabilities is violated, he or she shall have rights to require the relevant departments to deal with it, or apply to the arbitrate institution for arbitration, or litigate according to law.

[35]

Looking closely at the two provisions cited above, two limitations indicate their low effectiveness.

One limitation is the provision of “DPOs”. Article 59 and Article 60 have entitled persons with disabilities to protect their rights through DPOs, which means that DPOs can represent individuals with disabilities to protect their rights. In China, the DPOs can be categorized into two groups. One group includes civil organizations that purely focus on service provisions for persons with disabilities and their family members. Another group is the China Disabled Persons’ Federation at the national government level and its branches at provincial, municipal, county and town/district governmental levels. The Disabled Persons’ Federations countrywide are all semi-governmental institutions. The functions of these organizations include: (a) Represent the interests of persons with disabilities in China and help protect their legitimate rights; (b) Provide comprehensive and effective services to persons with disabilities; (c) Supervise affairs related to persons with disabilities commissioned by the Chinese government [45]. In fact, only the Disabled Persons’ Federations at each level can play the role of helping persons with disabilities protect their rights. However, at least two main reasons discourage these organizations to do so. On the one hand, since the Disabled Persons’ Federation system was established in 1986, the tendencies of “administrativization”, formalism and bureaucratization have become increasingly prominent, and they prefer regarding themselves as governmental organizations to the representative organization or service providers in practice. On the other hand, the organizational capacities of these organizations are weak. Although they strive for more governmental power, the semi-governmental nature determines that they are located in a disadvantaged position in the government system. One positive trend in the past five years is that in the national institutional reform context, the China Disabled Persons’ Federation is seeking to strengthen the representative and service functions, while weakening the governmental functions in reform [46].

Another limitation is the provision that refers to “relevant departments”. Article 59 makes evident that even if the Disabled Persons’ Federations hope to help individuals with disabilities protect their rights and interests, there is not explicit implementation guidance to determine what is the “relevant department”. It is not easy to determine the defendants of lawsuits when sending applications to the courts by persons with disabilities. Therefore, the rights enforcement mechanisms do not work barely at all in practice.

## 4. The Weak Impact of the Employment Quota Scheme Legal Framework

The concluding observations in the initial report on China by the CRPD Committee argues that the employment quota scheme is only of symbolic value due to deep-rooted discrimination. In fact, discrimination is just one aspect. The conflicts between the mainstream labor market and the employment quota scheme legal frameworks largely encourage employers to pay penalties rather than employ persons with disabilities. In this section, the evolutionary process of China’s employment quota scheme legal framework is first examined. Then, the reasons why the employment quota scheme’s legal framework does not work well are analyzed.

### 4.1. The Evolutionary Process of China’s Employment Quota Scheme Legal Framework

The employment quota scheme was adopted by the Law of the PRC on the Protection of Persons with Disabilities in 1990 (Order No. 36 [1990] of the President). Article 30 of this law stipulated:

A state organ, social group, enterprise, public institution or private non-enterprise entity shall arrange employment of persons with disabilities in a prescribed proportion and choose proper types of work and posts for them. If the prescribed proportion was not reached, it shall fulfill the obligation to ensure the employment of the disabled under the relevant provisions of the state. The state shall encourage the entity employers to arrange employment of persons with disabilities in excess of the prescribed proportion.

[26]

According to this provision, local governments should formulate corresponding measures for the implementation of the Law of the PRC on the Protection of Persons with Disabilities (Order No. 36 [1990] of the President) in order to implement the quota scheme. In this context, some local governments established regulations that employers who failed to meet the prescribed proportion were required to pay money to the disability employment security fund as an alternative.

In 1995, based on the local implementing experience, the Ministry of Finance promulgated the Notice of the Interim Measures for Management of the Disability Employment Security Fund (No. 5 [1995] of the Ministry of Finance) to regulate collection and payment of the fund on a national level. Article 2 of the Interim Measures stated that the contribution amount made by an employer was calculated as follows:

Payable amount = (number of total employees in the previous year × employment quotas required by the local government—number of employees with disabilities) × local average annual social wage in the previous year.

[47]

In 2007, China passed the Regulation of the PRC on the Employment of Persons with Disabilities (Order No. 488 [2007] of the State Council). Article 8 states that an employer shall hire disabled employees at a proportion of no less than 1.5% of the total employees. This was the first time that the quota was clearly specified. In 2008, the Law of the PRC on the Protection of Persons with Disabilities of 1990 (Order No. 36 [1990] of the President) was amended. Article 33 of the 2008 Amendment (Order No. 3 [2008] of the President) clearly expresses that the quota scheme is formally established as a national law system [34].

In 2015, the Ministry of Finance, the State Tax Bureau and China Disabled Persons’ Federation jointly issued the Measures for the Administration of Collection and Payment of the Disability Employment Security Fund (No. 72 [2015] of the Ministry of Finance and the State Tax Bureau). At the same time, the Notice of the Interim Measures for Management of the Disability Employment Security Fund (No. 5 [1995] of the Ministry of Finance) was abolished. These measures stated that the provincial (including autonomous region and centrally administered municipality) governments should establish their own measures based on the national measures. All employers in China should make contributions to the Disability Employment Security Fund if they do not meet the required number of disabled employees. Specifically, there were two main changes compared to the former Measures of 1995.

First, the calculation formula of the contribution amount was changed. Currently, if an employer fails to hire the locally required percentage of persons with disabilities, it is obliged to pay contributions to the Disability Employment Security Fund. Compared to the former calculation formula, the new one replaces “the local average annual social wage in the previous year” with “average annual wage of the employees in the previous year”. This means the penalties for employers with high average annual wage of the employees, such as the high technology sector, are substantially increased. The new calculation formula of the payable amount is below:

Payable amount = (number of total employees in the previous year × employment quotas required by the local government − number of employees with disabilities) × average annual wage of the employees in the previous year.

In addition, the collection agencies of the Disability Employment Security Fund have changed. It is now collected monthly by local tax bureaus, instead of employment service agencies affiliated with the China Disabled Persons’ Federation as was done previously. This change makes the collection more enforceable and avoids a lot of payment refusals and arrears. In practice, it is collected together with the social insurance contributions either once a month or in a lump sum once a year, subject to the practice of different locations [47].

### 4.2. Reasons that the Quota Scheme Legal Framework Is Not Well-Functioning

The employment relations regarding employers and employees with disabilities are regulated both by the mainstream labor market and the quota scheme legal frameworks. However, in China, conflicts between the two systems have led to employers paying the penalties rather than hiring persons with disabilities. In order to explore how the conflicts crowd out employment of persons with disabilities, the mainstream labor market legal framework is first outlined and then the protection effects of this framework are analyzed. Finally, the conflicting provisos between the mainstream labor market legal framework and the quota scheme legal framework which crowd out employment of persons with disabilities are described in detail.

#### 4.2.1. Legal Framework of the Mainstream Labor Market

There are three key laws in the mainstream labor market: the Labor Law of the PRC (Order No. 3 [1994] of the President) passed in 1994, the Labor Contract Law of the PRC (Order No. 65 [2007] of the President) adopted in 2007 which was first amended in 2012, and the Labor Dispute Mediation and Arbitration Law of the PRC (Order No. 80 [2007] of the President) promulgated in 2007.

The 1994 Labor Law of the PRC (Order No. 3 [1994] of the President) was the first market-oriented labor legislation after China transformed from a planned economy to a market economy. It mainly aims to construct a labor law framework and indicate that the labor relations of a market economy have been established [48]. This law only includes one hundred and six articles without other specific implementation guidance, so it was nearly ineffective in practice [49]. In 1995, the Labor Law Enforcement Inspection Report conducted by the Standing Committee of the National People’s Congress (the State legislature) concluded that employees’ rights were still being seriously violated, so the Committee began to discuss formulating the labor contract law in 2000. There was a fierce debate regarding either preferring protection to employees, or balancing protection between employers and employees for several years. Finally, since the employers were stronger than the employees, the legislative purpose of the 2007 Labor Contract Law of the PRC (Order No. 65 [2007] of the President) adopted preferring protection to employees.

To some extent, with the implementation of this law, the cases of the infringement upon the rights and interests of employees have declined, indicating the legislative purpose was achieved [50]. However, the law also has incurred strong criticism in two aspects: one is that the implementation of this law has increased labor costs and decreased employment flexibility [51]; another is that the regulations on labor dispatch have led to a sharp increase in the number of dispatched employees [52]. In 2007, the Labor Dispute Mediation and Arbitration Law of the PRC (Order No. 80 [2007] of the President) was passed and introduced the “Single Ruling System” principle, which is still based on the thinking of preferring protection to employees. The “Single Ruling System” refers to the practice where employees who disagree with the result of labor dispute arbitration can continue to apply for arbitration with no substantive and procedural restriction. However, the employers have no such rights as the employees. This principle further improves the power of employees in the employment relations, and consequently, employers have become more prudent in signing regular labor contracts with employees.

To conclude, the mainstream labor market legal system has two prominent protection effects: strictness of employment protection for regular employees and encouragement of employing dispatched employees. Both are analyzed in detail below.

#### 4.2.2. Protection Effects of the Mainstream Labor Market Legal Framework

**Figure 1:**
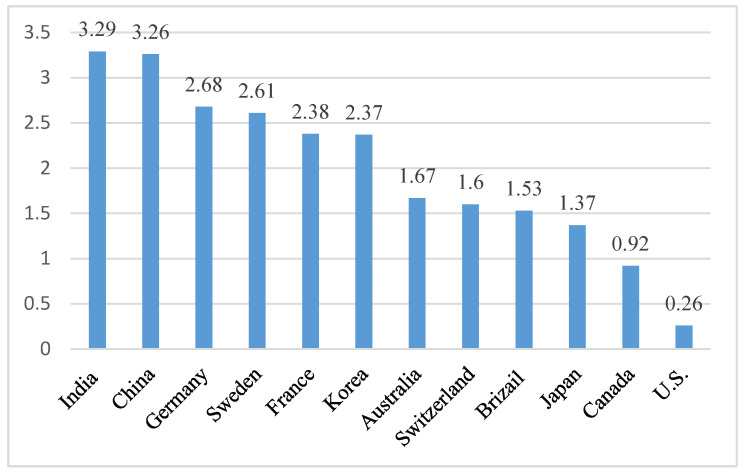
The OECD indicators of the strictness of employment protection-individual and collective dismissals in 2012 (regular contracts). Source: OECD. Indicators of the Strictness of Employment Protection—Individual and Collective Dismissals in 2012 (Regular Contracts). OECD: Paris, France, 2012. Available online: https://stats.oecd.org/Index.aspx?DataSetCode=EPL_OV (accessed on 2 August 2018) [54].

The first protection effect of the mainstream labor market legal system is the strict employment protection for regular employees. With the legislative purpose of “preferring protection to employees”, the Labor Contract Law (2012 Amendment) (Order No. 73 [2012] of the President) strictly limits the arbitrary dismissal of employees, leading to substantial increase in labor costs and decrease in employment flexibility for the employers. The specific provisions are expressed in Article 14 and Article 82. Article 14 describes how to renew and conclude a non-fixed term contract. Article 82 regulates the penalty if an employer does not sign a non-fixed-term labor contract with the employee [53]. According to the OECD indicators of the strictness of employment protection—the individual and collective dismissals (regular contracts) in 2012, the dismissal protection for regular contracts in China is much stricter than many developed countries (see Figure 1).

Another important factor contributing to the labor cost increase is the social insurance contributions. According to the Labor Contract Law (2012 Amendment) (Order No. 73 [2012] of the President), employers must pay social insurance (pension, health insurance, work injury, unemployment and maternity) contributions for regular employees. In addition, many employers provide the housing provident fund for employees with regular contracts. Many agree that the social insurance and housing provident fund contributions in China have become a great burden for the employers. According to a 2012 report in the official newspaper of the Central Committee of the Communist Party of China, People’s Daily, although China was not the country with the highest social insurance contributions in the world, it was still quite high (the social insurance contributions in selected countries are shown in Table 1) [55].

The second protection effect of the mainstream labor market legal system is encouragement of employing dispatched employees. The Labor Contract Law is also strongly criticized for introducing the concept and practice of labor dispatch employment. The term “labor dispatch” is described by the International Labor Organization (ILO) as “the practice of hiring employees through an employment service agency as opposed to the direct employment” [56]. China’s Ministry of Human Resources and Social Security defines “labor dispatch” as “a method of employment whereby the employer dispatches the employees it recruits to other employers, and the latter employers directly manage the working process of such employees” [57]. With the implementation of this law, the labor market has experienced a sharp rise in the number of dispatched employees. The core element of the labor dispatch employment relationship is “unequal pay for equal work” which encourage employers to employ as many dispatched employees as possible, instead of regular contracted workers. Although the Amendment of Labor Contract Law in 2012 advocates “equal pay for equal work” to address the overuse of dispatched workers, it has turned out to have limited effectiveness due to a lack of implementation and responsibility mechanisms [58]. For employers, it is more advantageous to opt for dispatched employees than regular contract employees because of the labor costs and dismissal flexibility. According to the official statistics in China, there were 20 million dispatched employees before the Labor Contract Law came into effect on 1 January 2008. However, it had increased to 60 million by the end of 2010, accounting for 20% of the total employees [59].

In addition, the “Single Ruling System” introduced by the Labor Dispute Mediation and Arbitration Law in 2007 prompts employers further to choose dispatched employees. The aim of the creation of this principle was to prevent an employer’s malicious litigation and the employee’s time-consuming rights protection efforts [60], which fully represents the legislative concept of preferring protection to employees [61]. However, there are more criticisms of the system than in favor. Some people criticize this system, saying that it is far removed from the basic meaning of “preferring protection to employees” which is upheld in the Labor Contract Law, because it is at the expense of restricting the litigation rights of employers [62].

#### 4.2.3. Conflicting Provisions between the General and Special Legal Frameworks

Summarizing the discussion previously, we can see that the mainstream labor market legal framework gives different protections for regular employees and dispatched employees. Specifically, for the employees with a regular labor contract, they can be protected in three ways. One is by increasing the dismissal strictness for regular employees through the provision of a non-fixed term labor contract. Another is for employers to pay social insurance contributions (most of the employers also must pay the housing provident fund) for the regular employees who have signed labor contracts. The third is that when a labor dispute occurs, the “single ruling system” means only the employee could continue to litigate while the employer has no such rights. For the dispatched employees, the “unequal pay for equal work” concept was introduced in the initial Labor Contract Law in 2007. Although the 2012 Amendment regulates “equal pay for equal work”, it is just on paper rather than really working in practice due to lack of implementation regulations. Therefore, employers are encouraged to hire as many dispatched employees as possible, instead of employees with regular labor contracts.

When employers fulfill the obligations of the employment quota scheme, there are two choices: to employ persons with disabilities, or to pay money to the Disability Employment Security Fund. However, the legal provisions of the quota scheme are exactly in conflict with the effects of the mainstream labor market legal framework, namely strictness of employment protection for regular employees and encouragement of employing dispatched employees. Specifically, there are three conflicts between the two legal frameworks that lead to excluding persons with disabilities from the labor market.

The first conflict is the identification criteria of an employee with disabilities. According to the Measures for the Administration of Collection and Payment of the Disability Employment Security Fund (No. 72 [2015] of the Ministry of Finance and the State Tax Bureau), an employee who is identified as employed in the quota scheme should meet these requirements: the employer shall award a contract of no less than one year, pay wages no less than the local minimum wage, and pay social insurance and housing fund contributions duly and fully for the employee with a disabled person identification card.

One of the criteria for identifying an employee with disabilities is the regular labor contract. However, the Labor Contract Law has increased the labor cost and employment flexibility for employers through the provisions of strictness of dismissal and the social insurance and housing provident fund contributions, so employers are extremely inclined to sign labor contracts with so-called “high-quality employees”. For persons with disabilities, they have relatively lower level of education (see Table 2), so most of the employers prefer to pay the penalty rather than hire persons with disabilities.

The second conflict is that dispatched employees are counted into the number of the dispatch service providers. In order to address the overuse of labor dispatch, the 2012 Labor Contract Law Amendment regulates that “employers shall employ dispatched employees only in the temporary, auxiliary or substitute job positions” (Article 66) [53]. In 2014, the Interim Provisions on Labor Dispatch further provided that “an employer shall strictly control the number of dispatched workers. They shall be no more than 10% of the total employees in Article 4” [57]. However, the two restrictive articles still have limited effects of constraining the number of dispatched employees. In practice, the labor dispatch always appears as a form of business outsourcing and the dispatched employees are still very popular [64].

In terms of the quota scheme, Article 8 of the Measures for the Administration of Collection and Payment of the Disability Employment Security Fund (No. 72 [2015] of the Ministry of Finance and the State Tax Bureau) regulates dispatched employees are counted into the labor dispatch providers. Employers are encouraged to hire dispatched employees, because they are only in temporary, auxiliary or substitute job positions with low wages and poor employment protections. According to the Disability Employment Security Fund calculation formula, if the average annual wages of the employees in the previous year are low, the contributions of the employers are also correspondingly low, so they opt to pay the penalty rather than hire persons with disabilities.

The third conflict is the special regulations on labor protection, workplace accommodation and vocational training for employees with disabilities, which further “crowds out” their opportunities of employment. In the Law of the PRC on Protection of Persons with Disabilities (2008 Amendment) (Order No. 3 [2008] of the President), Article 38 Paragraph 3 stipulates that the employers shall provide the reasonable working accommodation, and protection, equipment and living facilities and modify the working place for the disabled employees. Article 39 of this law regulates that employers shall provide on-the-job technical training for disabled employees to enhance their work skills.

In the Regulation on Employment of Persons with Disabilities (Order No. 488 [2007] of the State Council), Article 13 states that the employer shall provide disabled employees with labor accommodations and protection suitable for their health condition and uphold the reasonable accommodations principle. Article 14 provides that the employer shall offer pre-job training, on-the-job training and job transfer according to the actual situation of the disabled employees.

These special provisions for disabled employees increase the cost to employers and thus disputes easily arise. Once labor contracts are signed, persons with disabilities are always in an advantaged position in case of a labor dispute occurs. Moreover, the “Single Ruling System” in the Labor Dispute Mediation and Arbitration Law is not friendly to the employers.

The conflicts between the mainstream labor market and quota scheme legal provisions are summarized in Table 3.

According to the analysis above, it can be concluded that the conflicts between the mainstream labor market legal framework and the employment quota scheme legal frameworks have the “crowding out effect” of disability employment. This is demonstrated by the number of employees employed in the quota scheme and the increasing amounts of the fund recent years.

**Figure 2:**
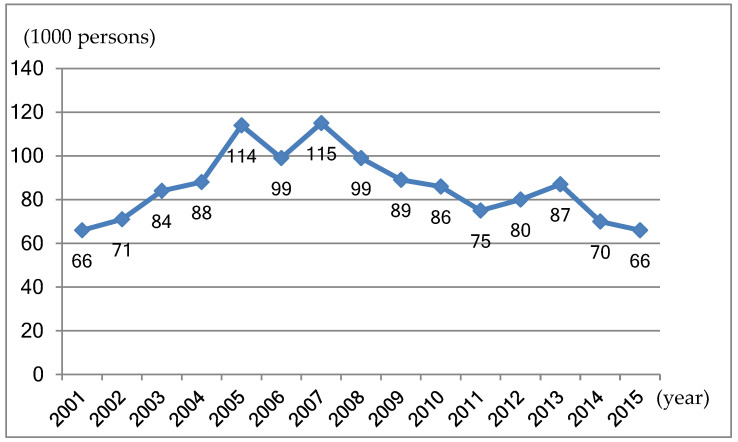
The number of annual new employed individuals with disabilities in urban China areas (2001–2015) Source: China’s Disabled Persons Federation (CDPF). The Disability Cause Development Statistical Communiqué in 2001–2015; CDPF: Beijing, China. Available online: http://www.cdpf.org.cn/sjzx/tjgb/ (accessed on 20 January 2020) [65]. Notes: Before 2016, the employment statistic of disability employment was separated in urban and rural areas, and the number of employees in quota scheme was counted in the urban areas. Since 2016, there is no longer a urban-rural divide. Therefore, the old data is relatively clearer to show the trend of the employment offered in the quota scheme, and here the old data is adopted.

Between 2001 and 2015, the annual new number of quotas scheme employees with disabilities decreased sharply since 2007 (see Figure 2).

**Figure 3:**
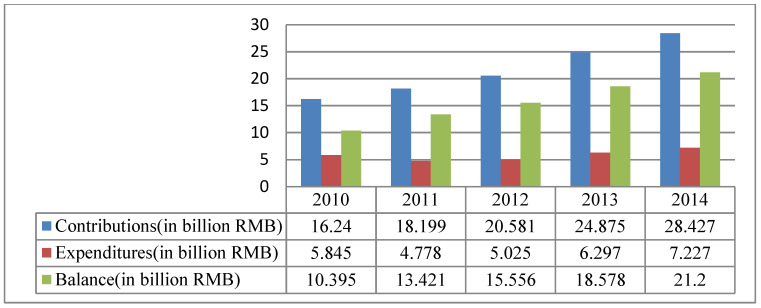
The Contributions, Expenditures and Balance of the Disability Employment Security Fund (2010–2014). Source: Ministry of Finance. Ministry of Finance Annual Statistic in 2010–2014. Ministry of Finance: Beijing, China. Available online: http://www.mof.gov.cn/index.htm (accessed on 3 February 2020) [66].

While the number of employed persons with disabilities in the quota scheme is in a downward trend, the available data (the only public data is from 2010 to 2014) shows that the amount of the Disability Employment Security Fund is increasingly growing and there is large balance in this fund (see Figure 3).

## 5. Conclusions

In many countries, disability policies have been layered on top of each other. The thinking about disability has undergone a dynamic process of change. As new paradigms of disability have gained acknowledgement, and new policies have been adopted, while the preexisting policies have not been replaced or revised in many cases. As a result, the strategy as a coherent whole often has failed to be developed due to the conflicts and contradictions in the policies. Further, this layering is also partly because the new policies cannot fully meet the needs that earlier policies have addressed.

In terms of disability employment policies, choices between anti-discrimination legislation and employment quota schemes largely seem to be based on cultural differences, attitudes and experience [67]. Anti-discrimination legislation is represented in Anglo-Saxon countries (e.g., USA, UK) and the employment quota scheme is the main plank of disability employment in Western Europe [68]. However, the tradition has been changing and international development in disability policy clearly tends towards anti-discrimination legislation [69]. Large numbers of countries have superimposed anti-discrimination policies on their pre-existing employment quota schemes, despite the fact that they are based on different views of disability. The employment quota scheme is based on the medical model of disability which assumes that the difficulties that persons with disabilities face in the labor market are from their own inability. In contrast, anti-discrimination legislation is inspired by the social model of disability. Such an approach focuses on the competence and capacity of persons with disabilities and related policies aim to remove the inequality and discrimination created by society.

The practice of disability employment demonstrates that neither the anti-discrimination policy nor the employment quota scheme can sufficiently address the difficulties that persons with disabilities encounter in the open labor market. For the employment quota scheme, evidence from different countries has shown that the outcomes of three main forms-legislative recommendation, legislative obligation with no effective sanctions, and legislative obligation backed up by sanctions—are limited in promoting employment of persons with disabilities. Under the legislative recommendation approach, employers are asked to voluntarily employ persons with disabilities without any sanction. Such an approach has little impact on the employment of persons with disabilities in the labor market. The legislative obligation with no effective sanctions means that the employers have the legal obligation to employ persons with disabilities, but there is no effective sanction forcing them to comply with the law. The quota scheme in the UK is typical of this type. In 1996, the UK abolished the quota scheme due to its ineffectiveness [70]. The third form of quota scheme is the legislative obligation backed up by sanctions, which originates from Germany. Under this form, employers are obliged to either employ certain quotas of persons with disabilities or pay a penalty. The quota scheme in China is fits into this approach. This form is regarded as the most effective way to promote employment of persons with disabilities. However, the practice in Germany shows that its effectiveness has been gradually weakened over the years [71]. The anti-discrimination legislation is also limited in removing discrimination due to many factors such as the definitions of disability, discrimination, and reasonable accommodations [72].

At present, the human rights model espoused by the CRPD is widely acknowledged to offer a way of thinking and a tool to develop a more coherent and sustainable disability strategy to enhance the effectiveness of various policies. The CRPD reaffirms all human rights for all persons with disabilities and motivates the policies based on principles of respect for human dignity. Thus, the quota scheme and the anti-discrimination legal framework both are complementary components of respect for human dignity of persons with disabilities.

In China, the employment quota scheme and anti-discrimination legal frameworks are part of the disability policies. However, as analysis in this article has shown, both of them have shortcomings that result in limited outcomes for promoting employment of persons with disabilities. For the anti-discrimination framework, the lack of a definition of disability, the missing definition of discrimination, and the absence of effective enforcement mechanisms have led the anti-discrimination legal framework to be nearly ineffective in practice. For the employment quota scheme, the employers are encouraged to pay a penalty rather than hire persons with disabilities due to the conflicts between the mainstream labor market legal framework and the quota scheme legal framework. In the long history of China, the views that persons with disabilities should be cared for by society have deeply influenced policy making. However, the traditional strong government can be helpful for implementing some policies relatively quickly. In the short term, on the one hand, China should strength the employment quota scheme by amending the conflicting provisions in the legal framework. This is not only helpful for promoting the employment of persons with disabilities, but also for raising public awareness about this issue. On the other hand, China should improve the outcomes of anti-discrimination legislation by clearly defining disability and discrimination, and improving the enforcement mechanisms. Meanwhile, although China ratified the CRPD in 2008, it has not obviously affected the disability policies. Thus, the CPRD should be emphasized more strongly, including using a human rights model instead of the medical model to develop a coherent and sustainable disability legal framework for promoting participation of persons with disabilities, rather than focusing on seeing them as passive recipients of care in the long run.

## Figures and Tables

**Table 1:** The social insurance contribution rates in selected countries.

Country	Employee (%)	Employer (%)	Total (%)
France	9.9	32.68	42.58
Germany	20.43	20.85	41.28
Italy	9.19	31.78	40.97
Poland	22.71	17.38	40.09
China	11	29	40
Belgium	13.07	24.8	37.87
Spain	6.25	31.08	37.33
India	13.75	22.36	36.11
Russia	0	30.2	30.2
Brazil	8	21	29
Sweden	7	20.92	27.92
Japan	13.12	13.77	26.89
U.S.	7.65	9.7	17.35
South Korea	7.79	8.74	16.53
Canada	6.73	7.44	14.17
Mexico	2	8.6	10.6
Thailand	5	5.2	10.2
Indonesia	2	7.24	9.24

Source: People’s Daily. The Social Insurance Contribution is not the Highest in the World in China: Social Security and Pension Investigation; People’s Daily: Beijing, China, 11 September 2012 (Page 4).

**Table 2:** The education level of persons with disabilities aged 18 and above in China (2007–2013).

Year	2007	2008	2009	2010	2011	2012	2013
	Percent of the Total Persons with Disabilities						
Illiterate	42.4	42.1	41.8	40.9	37.7	36.9	36.3
Primary School	35.1	35.0	34.8	35.2	36.9	37.6	38.0
Junior Middle School (Secondary School)	15.8	15.9	16.5	16.7	18.0	18.2	18.4
Senior Middle School (High School)	3.9	4.0	4.1	4.3	4.4	4.5	4.3
Technical Secondary School	1.5	1.5	1.5	1.5	1.4	1.3	1.4
Junior College	0.8	1.0	0.9	1.0	1.0	1.0	1.1
Undergraduate and Above	0.5	0.5	0.5	0.5	0.5	0.5	0.5

Source: China Disabled Persons’ Federation (CDPF). The 2013 Disability Status and Wellbeing Process Monitoring Report; CDPF: Beijing, China, 2014. Available online: http://www.cdpf.org.cn/sjzx/jcbg/201,408/t20140812_411000.shtml (accessed on 25 January 2020) [63].

**Table 3:** The conflicting provisions between mainstream labor market and quota scheme legal frameworks.

Conflicts	Mainstream Labor Market	Quota Scheme
Labor contract	Avoid signing labor contract	Sign a labor contract with the disabled person for a term of at least one year.
The calculation of the total employees	Prefer dispatched employees	Dispatched employees are included in the calculation of the total employees of employment service agencies.
Social insurance and housing provident fund contributions	High labor cost for the employers	Pay social insurance and housing fund contributions duly and fully.
Single Ruling System	Preferring protection for employees	Special labor protection and workplace condition (reasonable accommodations)

Source: Authors’ own compilation.

## References

[1] World Health Organization (WHO), World Bank (2011). The World Report on Disability.

[2] Cheng K. (2018). Uphold the targeted poverty alleviation strategy, focus on addressing the poverty caused by disease. Adm. Reform.

[3] Buckup S. (2019). The Price of Exclusion: The Economic Consequences of Excluding People with Disabilities from the World of Work.

[4] The China Disabled Persons’ Federation (CDPF) (2019). The Disability Cause Development Statistical Communiqué in 2019.

[5] Lai D.S., Liao J., Liu W. (2008). Analysis of the employment and its influencing factors of persons with disabilities in China. J. Renmin Univ. China.

[6] Lv X.J., Zhao M.M. (2012). Analysis of the economic growth effects on employment of persons with disabilities. Hubei Soc. Sci..

[7] Liao H.Q. (2014). Exchanging, welfare or disincentive—Protective employment of persons with disabilities. Sociol. Stud..

[8] Jones M., Marks L.A.B., Jones M., Marks L.A.B. (1999). Law and the social construction of disability. Disability, Diversity and Legal Change.

[9] Kanter A.S. (2011). The law: What’s disability studies got to do with it or an introduction to disability legal studies. Columbia Hum. Rights Law Rev..

[10] Liachowitz C.H. (1988). Disability as a Social Construct: Legislative Roots.

[11] Humpage L. (2007). Models of disability, work and welfare in Australia. Soc. Policy Adm..

[12] Linker B. (2013). On the borderland of medical and disability history: A survey of the fields. Bull. Hist. Med..

[13] Brandon T., Pritchard G.W. (2011). Being fat: A conceptual analysis using three models of disability. Disabil. Soc..

[14] Bury M.R., Wood P.H.N. (2009). Sociological perspectives in research on disablement. Disabil. Rehabil..

[15] World Health Organization (WHO) (1980). International Classification of Impairments, Disabilities, and Handicaps: A Manual of Classification Related to the Consequence of Disease.

[16] Oliver M.J. (1990). The Politics of Disablement.

[17] Crow L., Barnes C., Mercer G. (1996). Including all of our lives: Renewing the social model of disability. Exploring the Divide: Illness and Disability.

[18] Hahn H. (1985). Towards a politics of disability: Definitions, disciplines, and policies. Soc. Sci. J..

[19] Degener T., Fina V.D., Cera R., Palmisano G. (2017). A new human rights model of disability. The United Nations Convention on the Rights of Persons.

[20] Degener T., Quinn G., Yee S., Breslin M.L. (2002). A survey of international, comparative and regional disability law reform. Disability Rights Law and Policy: International and National Perspectives.

[21] Shakespeare T., Watson N., Barnartt S.N., Atlman B.M. (2001). The social model of disability: An outdated ideology?. Exploring Theories and Expanding Methodologies: Where We Are and Where We Need to Go (Research in Social Science & Disability):2(Research in Social Science and Disability).

[22] World Health Organization (WHO) (2001). International Classification of Functioning, Disability and Health: ICF.

[23] UN (2006). The United Nations Convention on the Rights of Persons with Disabilities (CRPD).

[24] Gao H.R. (2019). Connotations and implications of people’s livelihood. J. Xiamen Univ. (Arts Soc. Sci.).

[25] Lu D.Y. (1986). Disability History in China.

[26] Standing Committee of the National People’s Congress (1990). Law of the People’s Republic of China on the Protection of Persons with Disabilities (Order No.36[1990] of the President).

[27] China Disabled Persons’ Federation (CDPF) (1995). Notice of the China Disabled Persons’ Federation on the Unified Issue of the “China Disability Certification Card” (CDPF, No.61 [1995]).

[28] Harris S.P., Owen R., Gould R. (2012). Parity of participation in liberal welfare states: Human rights, neoliberalism, disability and employment. Disabil. Soc..

[29] Böheim R., Leoni T. (2018). Sickness and disability policies: Reform paths in OECD countries between 1990 and 2014. Int. J. Soc. Welf..

[30] Thornton P., Lunt N. (1994). Disability and employment: Toward an understanding of discourse and policy. Disabil. Soc..

[31] Waddington L., Degener T., Koster-Dreese Y. (1995). A European right to employment for disabled people. Human Rights and Disabled Persons: Essays and Relevant Human Rights Instruments.

[32] Diller M. (1998). Dissonant disability policies: The tensions between the Americans with Disabilities Act and federal disability benefit programs. Tex. Law Rev..

[33] Committee on the Rights of Persons with Disabilities (2012). Concluding Observations on the Initial Report of China (no.41).

[34] Standing Committee of the National People’s Congress (2008). Law of the PRC on the Protection of Persons with disabilities (2008 Amendment) (Order No.3 [2008] of the President).

[35] State Council (2007). Regulation of the PRC on the Employment of Persons with Disabilities (Order No.488 [2007] of the State Council).

[36] Degener T. (2006). The definition of disability in German and Foreign Discrimination Law. Disabil. Stud. Q..

[37] Degener T., Lawson A., Gooding C. (2005). Disability discrimination law: A global comparative approach. Disability Rights in Europe: From Theory to Practice.

[38] Altman B.M., Albrechat G.L., Seelman K.D., Bury M. (2001). Disability definitions, models, classification schemes and applications. Handbook of Disability Studies.

[39] Ministry of Human Resources and Social Security of the People’s Republic of China (2005). The General Standard for Civil. Service Recruitment Health Examination.

[40] Beijing Newspaper (2017). Prohibiting Using the Health Examination Standard to Refuse Persons with Disabilities.

[41] Gooding C., Casserley C., Lawson A., Gooding C. (2005). Open for all? Disability discrimination laws in Europe relating to goods and services. Disability Rights in Europe: From Theory to Practice.

[42] Curran L. (2019). Such as in the disability discriminatory legislation of Australian and UK, the “direct discrimination”, “indirect discrimination” and “reasonable accommodation” are all clearly defined. Legal Rights and Protection of People with Disabilities in the Workplace: Australia, Austria, Canada, France, Germany, Italy, Netherlands, New Zealand, Norway, Spain, Sweden, United Kingdom, United States.

[43] The United States Congress (2008). ADA AMENDMENTS ACT OF 2008(PL 110-325 (S 3406)).

[44] (2016). The Legal Daily is a China state-owned newspaper under the supervision of the Central Commission for Political and Legal Affairs that is published in China and primarily covers legal developments. It’s the Time to Introduce an Employment Anti-Discrimination Law.

[45] China Disabled Persons’ Federation (CDPF) (1990). Main Functions of the China Disabled Persons’ Federation.

[46] China Disabled Persons’ Federation (CDPF) (2018). Zhou Changkui: Thirty Years of China Disabled Persons’ Federation, Soaring again in Refreshing Reform.

[47] Ministry of Finance, State Tax Bureau, China Disabled Persons’ Federation (2015). Measures for the Administration of Collection and Payment of the Disability Employment Security Fund (No.72 [2015] of the Ministry of Finance and the State Tax Bureau).

[48] Wang Q.X., Shi C. (2020). Reviews and reflections on 70 years labor law in New China. Seeker.

[49] Lin J., Deng J. (2009). The paradigm transformation of the labor law in China. Politics Laws.

[50] Cheng Y.Y. (2007). The Contract Law: Constructing and developing the harmonious and stable Labor relations. J. Renmin Univ. China.

[51] Dong B.H. (2016). The ten unbalanced issues of the China Labor Contract Law. Explor. Free Views.

[52] Zheng S.Y. (2014). The balance between control and relaxation of dispatched employees in China—Studies on the Article 58 Paragraph 2 of the Labor Contract Law of China. Law Sci..

[53] Standing Committee of the National People’s Congress (2012). Labor Contract Law (2012 Amendment) (Order No.73 [2012] of the President).

[54] OECD (2012). Indicators of the Strictness of Employment Protection—Individual and Collective Dismissals in 2012 (Regular Contracts).

[55] People’s Daily (2012). China’s Social Security Contribution Rate is not the Highest in the World: Social Security and Pension Insurance Survey.

[56] Liu G.H. (2014). Private Employment Agencies and Labor Dispatch in China.

[57] Ministry of Human Resources and Social Security (2014). Interim Provisions on Labor Dispatch.

[58] Xie Z.Y. (2015). The reasons and resolutions of the regulatory failure of labor dispatch. Glob. Law Rev..

[59] Jiang Y.Z. (2011). Authoritative Report States That “Dispatched Employees” Reaches 60 Million and the National Federation of Trade Unions Proposes to Amend the Labor Contract Law.

[60] Lv W.Z. (2010). Studies on the several issues of “Single Ruling System” in labor dispute. J. Shandong Trial.

[61] Hong D.Y. (2008). Comments on the Labor Dispute Mediation and Arbitration Law. Acad. Bimest..

[62] Xie Z.Y. (2008). The concept, system and challenge of addressing the labor disputes in China. Chin. J. Law.

[63] China Disabled Persons’ Federation (CDPF) (2014). The 2013 Disability Status and Wellbeing Process Monitoring Report.

[64] Qiu J. (2018). Tenth anniversary of the Labor Contract Law series (eleventh): Research on the labor dispatch in the “labor Contract Law”. China Labor.

[65] China’s Disabled Persons Federation (CDPF) (2014). The Disability Cause Development Statistical Communiqué in 2001–2015.

[66] Ministry of Finance (2014). Ministry of Finance. Ministry of Finance Annual Statistic in 2010–2014.

[67] OECD (2003). Transforming Disability to Ability: Policies to Promote Work and Income Security for Disability People.

[68] Waddington L., Diller M. (2002). Tensions and coherence in disability policy: The uneasy relationship between social welfare and civil rights models of disability in American, European and international employment law Symposium. Principles to Practice: Disability Rights Law and Policy International and National Perspectives.

[69] Priestley M., Castles F.G., Leibfried S., Lewis J., Obinger H., Pierson C. (2010). Disability. Oxford Handbook of the Welfare State.

[70] Woodhams C., Corby S. (2007). Then and now: Disability legislation and employers’ practice in the UK. Br. J. Ind. Relat..

[71] Fuchs M. (2014). Quota Systems for Disabled Persons: Parameters, Aspects, Effectivity.

[72] Curran J., Benard M., Dycker S.D., Fina V.D., Kühnel V., Mayr M., Nadakavukaren K. (2019). Legal Rights and Protection of People with Disabilities in the Workplace: Australia, Austria, Canada, France, Germany, Italy, The Netherlands, New Zealand, Norway, Spain, Sweden, United Kingdom, United States.

